# Development and validation of novel models based on clonality immunoglobulin gene rearrangement for evaluation of bone marrow involvement and prognostic prediction in patients with diffuse large B-cell Lymphoma: a multicenter retrospective study

**DOI:** 10.3389/fimmu.2025.1547056

**Published:** 2025-04-14

**Authors:** Zelin Liu, Qianping Li, Ruiqi Liu, Yangyang Ding, Ya Liao, Linhui Hu, Lianfang Pu, Chunhua Zhu, Shenghui Zhang, Shudao Xiong

**Affiliations:** ^1^ Hematological Lab, The Second Affiliated Hospital of Anhui Medical University, Hefei, Anhui, China; ^2^ Department of Hematology, The Second Affiliated Hospital of Anhui Medical University, Hefei, Anhui, China; ^3^ Institute of Hematology, Wenzhou Medical University, Wenzhou, Zhejiang, China; ^4^ Department of Hematology, The First Affiliated Hospital of Wenzhou Medical University, Wenzhou, Zhejiang, China; ^5^ Department of Endocrinology, Qilu Hospital of Shandong University, Jinan, Shandong, China; ^6^ Air Force Health Care Center for Special Services, Hangzhou, Zhejiang, China; ^7^ Research Center for Translational Medicine, The Second Hospital of Anhui Medical University, Hefei, Anhui, China

**Keywords:** diffuse large B cell lymphoma, bone marrow involvement, immunoglobulin gene rearrangement, positron emission tomography-computed tomography, flow cytometry, bone marrow cytology, bone marrow biopsy pathology, prognostic model

## Abstract

**Introduction:**

Bone marrow involvement (BMI) is a poor prognostic factor in diffuse large B cell lymphoma (DLBCL), and accurate evaluation of BMI is crucial for determining stages and prognosis. This study aimed to identify the most effective examinations for evaluating BMI in DLBCL, including positron emission tomography-computed tomography (PET/CT), immunoglobulin gene rearrangement (IGR), flow cytometry (FCM), bone marrow cytology (BMC) and bone marrow biopsy pathology (BMB), and to further explore its prognostic significance in DLBCL patients.

**Methods:**

This retrospective study included 364 newly diagnosed DLBCL patients, all of whom underwent PET/CT, IGR, FCM, BMC, and BMB at diagnosis. Survival outcomes were analyzed via Kaplan-Meier and Cox regression models. Novel prognostic models incorporating combined IGR and BMB results were developed in a training cohort.

**Results:**

Compared to other detection methods, Clonal IGR BMI-positive were found the highest rate of 114 patients (31.3%), and IGR BM involvement-positive patients of DLBCL had the worst survival outcomes, especially among patients in stages I to III (P<0.001). Notably, PET/CT existed some limitations in BMI diagnosis, particularly in stage IV patients (P>0.05). Additionally, the combination of IGR and BMB demonstrated superior prognostic predictive capability for the patients in stage IV (PP<0.001). Multivariate analysis further confirmed that double-positive BMI of IGR and BMB was an independent prognostic factors of PFS (P=0.026) and OS (P=0.042). In addition, the novel IPI and NCCN-IPI stratification models were established by incorporating the combination of IGR and BMB in training group. The C-index of novel models were increased when IGR and BMB were supplemented in our cohort.

**Discussion:**

Our results suggest that IGR is the most valuable methods for evaluating BMI compared to traditional detection methods. Adding the combination of IGR and BMB to the IPI and NCCN-IPI score may improve their predictive ability. In summary, IGR is essental for evaluation of BMI and provide an ideal method for disease staging and risk stratification in DLBCL patients in the rituximab era.

## Introduction

Diffuse large B-cell lymphoma (DLBCL) is an aggressive neoplasm originating from mature B cells, representing the most prevalent subtype of non-Hodgkin lymphoma with a potential infiltration into the bone marrow, accounting for approximately 11%-25% of cases (1). In DLBCL, bone marrow involvement (BMI) holds clinical significance as it contributes to Ann Arbor staging and clinical risk stratification indexes, including the international prognostic index (IPI) and National Comprehensive Cancer Network-IPI (NCCN-IPI) (2).

Recently, positron emission tomography-computed tomography (PET/CT) has emerged as a pivotal tool for assessing staging, prognosis, and treatment efficacy (3). Numerous studies have demonstrated that PET/CT not only provides higher accuracy but also complements bone marrow biopsy pathology (BMB) in detecting bone marrow involvement in newly diagnosed DLBCL patients (4–6). However, due to inter-individual variations in basal metabolism, relying solely on specific standardized uptake value (SUV) thresholds to determine the positivity of PET/CT scans has significant limitations. Additionally, PET/CT presents challenges, including high cost and radiation risk for patients (7, 8). Therefore, it is crucial for clinicians to explore efficient and cost-effective approaches diagnostic approaches for assessing BMI in newly diagnosed patients.

Clonal immunoglobulin gene rearrangement (IGR) is a crucial mechanism in the tumorigenic B-cell malignant tumors and has garnered increasing attention for its role in the diagnosis and prognosis of B-cell lymphoma and leukemia (9). For instance, the detection of IGR aids in determining the lesion’s nature and distinguishing between benign and malignant lymphocytes (10, 11). As a specific marker for B-lymphocyte clones, IGR is instrumental in diagnosing B-cell lymphoma (12, 13).

Flow cytometry (FCM) and bone marrow aspiration cytology (BMC) are commonly used to diagnose BMI in patients with DLBCL. FCM determines the extent of infiltration by analyzing the immunophenotyping of tumor cells within the patient’s bone marrow (14, 15), while BMC was used to assess the degree of infiltration by microscopic examination of tumor cell size, morphology, and proportion (16). Although studies have not confirmed their ability to independently evaluate bone marrow infiltration in patients, these techniques are increasingly combined with molecular methods to enhance diagnosis and risk stratification. These combined methods offer significant guidance for prognostic assessment (16–18).

This multicenter retrospective study aimed to determine whether combining one or more auxiliary examinations could enhance the diagnostic efficacy for BMI compared to PET/CT in DLBCL patients receiving a rituximab-containing regimen (R-CHOP). Additionally, we sought to develop a novel model for predicting survival prognosis based on the results of multiple auxiliary examinations, including IGR, to facilitate early clinical risk stratification.

## Materials and methods

### Patients’ characteristics

A total of 364 patients with newly diagnosed DLBCL between February 2013 and August 2023 were enrolled from the Second Affiliated Hospital of Anhui Medical University, the First Affiliated Hospital of Wenzhou Medical University, and Qilu Hospital of Shandong University. This study was approved by the local institutional review board, and all participants provided written informed consent.

The inclusion criteria for patients in this cohort were as follows: a) aged 16 years or older with a confirmed diagnosis of DLBCL according to the 2016 World Health Organization (WHO) criteria; b) who underwent PET/CT, IGR, BMB, and BMC at diagnosis; c) without any malignancy other than lymphoma at the time of diagnosis; d) treated by rituximab-containing regimen.

Exclusion criteria included: a) patients with primary involvement of the central nervous system; b) DLBCL cases who had other cancer before; c) individuals with previous diagnosis and treatment at other hospitals; d) lacking complete information including age at diagnosis, sex, histology, Ann Arbor staging, IPI, NCCN-IPI, R-CHOP treatment regimen, date of relapse, date of death or documented date of last visit.

The classification of Ann Arbor stage IV at diagnosis was based on morphological bone marrow involvement (19). The date of death was obtained from clinical records or by telephone communication with the patient’s relatives.

### Bone marrow biopsy pathology

All patients included in the study had a bone marrow biopsy pathology (BMB), from which tissue samples (0.8-1.0 × 0.3 cm) were obtained from the posterior superior iliac spine. The samples were fixed in 10% formalin in PBS buffer (pH 7.2) for 24 hours. The samples were sectioned to a thickness of 2-5 microns and stained with the B-cell marker CD20 as well as T-cell markers CD3. Two experienced hematopathologists independently examined the samples for lymphoma involvement. A bone marrow infiltration rate of ≥30% with diffuse or interstitial patterns was considered indicative of high-volume of infiltration (7). Concordant BMI was defined as bone marrow involvement by predominantly large non-cleaved DLBCL cells, while discordant BMI was characterized by the presence of mainly small and low-grade lymphoma cells (2).

### PET/CT and image analysis

All newly diagnosed DLBCL patients in this cohort underwent ^18^F-FDG PET/CT examinations. The PET/CT images were reviewed by two experienced nuclear medicine physicians, who visually confirmed findings based on standardized uptake values (SUVs). Focal FDG uptake in bone marrow (BM) was defined as one or more regions where bone uptake exceeded liver activity but was lower than brain activity on PET images. Diffuse FDG uptake in BM was classified as heterogeneous uptake exceeding normal liver activity, without any focal lesions. Diffuse homogeneous FDG BM uptake related to benign conditions, such as inflammation or severe anemia, was excluded. Positive PET/CT findings were classified as PET BM involvement.

### IGR clonality detection and analysis

DNA was extracted from the bone marrow aspirates samples of cases with DLBCL, IGR clonality was examined by the using gene scanning through EuroClonality/BIOMED-2 guidelines (20), and the results were interpreted by experienced professional experimenters following the provided instructions (13). The positive results of IGR clonality were determined as IGR BMI.

### Flow cytometry immunophenotyping

The bone marrow samples were labeled with antibodies targeting CD45 and B-cell antigens, including CD19 and κ/λ light chains, and subsequently analyzed by flow cytometry. Abnormal cell populations were identified based on size and granularity using side scatter (SSC) gating in cells stained with CD45. More than 150,000 cells were analyzed using CellQuest software, with a positivity threshold of over 20% for each antigen considered indicative of abnormal infiltration. This data was used to determine the presence of lymphoma infiltration in the bone marrow, defined as FCM BM involvement.

### Morphologic analysis of bone marrow cells

The bone marrow smear was stained using Wright’s stain, and 200 nucleated cells were counted at the body-tail junction to determine the lymphocyte (or lymphoma cell) ratio. Morphological changes in lymphoid cells were meticulously examined throughout the smear to assess bone marrow involvement (BMI). The diagnostic criteria for lymphoma cell bone marrow involvement are defined as follows: on microscopic examination of bone marrow aspirate smears, if tumor cells constitute ≥5% of the total bone marrow cell population, bone marrow involvement is suggested. The final assessment was conducted and interpreted by two experienced hematological morphologists. Positive morphologic findings for BMI were classified as BMC BM involvement.

### Statistical analysis

Data analysis was performed using SPSS 25.0 and R software version 3.1.2. The chi-square test was applied for categorical variables, while the two-sided Student’s t-test was used for quantitative variables with a normal distribution. The relationship between BMI and clinical parameters was assessed using either the Spearman or Pearson correlation test, depending on data characteristics. The sensitivity and specificity of each method were compared against the reference standard of BMI from bone marrow biopsy pathology. Combinations of two diagnostic methods were evaluated using tandem testing to determine their collective sensitivity and specificity. Predictive efficacy was assessed using ROC analysis, and the Bayesian rule was applied to calculate the positive predictive value (PPV) and negative predictive value (NPV) for BMI tests. The primary endpoint of this study was progression-free survival (PFS), defined as the time from DLBCL diagnosis to disease progression, relapse, death, or last follow-up. The secondary endpoint was overall survival (OS), measured from diagnosis to death or last follow-up. Survival estimates were generated using the Kaplan-Meier method, with differences evaluated by the stratified log-rank test.

To develop and test the new risk stratification model, we randomly selected two-thirds of the patients as the training set (n=243) and allocated the remaining patients to the testing set (n=121). The hazard ratio (HR) and 95% confidence interval (CI) were estimated using the Cox regression model, with statistical significance set at P<0.05. The area under the curve (AUC) was used to compare the predictive efficacy of the risk stratification models. The C-index was calculated based on the individual NCCN-IPI value and age, followed by the inclusion of a combination of IGR and BMB (21).

## Results

### The characteristics of the patients with DLBCL in our cohort

All the 364 newly diagnosed DLBCL patients in this study, the median age was 59.8 years (range: 16-87 years). Of these patients, 73 patients (20.1%) were in stage I, 69 patients (19.0%) were in stage II, 50 patients (17.3%) were in stage III, and 172 patients (47.3%) were in stage IV. According to the IPI scoring system, 106 cases (29.1%) were classified as low-risk, 64 cases (17.6%) as medium-low risk, 104 cases (28.6%) as medium-high risk, and 90 cases (24.7%) as high-risk. According to the NCCN-IPI scoring system, 20 (5.5%), 144 (39.6%), 158 (43.4%), and 42 (11.5%) of patients were classified into low, low-intermediate, high-intermediate, and high risk groups, respectively. The additional clinical characteristics are summarized in **Table 1**.

**Table 1:** Clinical characteristics of DLBCL patients (n=364).

Characteristics	NO. (%)
Age, median (range)	59.8 (13-87)
Sex	
Male	190 (52.2)
Female	174 (47.8)
Stage at diagnosis	
I	73 (20.1)
II	69 (19.0)
III	50 (17.3)
IV	172 (47.3)
IPI score	
Low	106 (29.1)
Low-intermediate	64 (17.6)
High-intermediate	104 (28.6)
High	90 (24.7)
NCCN-IPI score	
Low	20 (5.5)
Low-intermediate	144 (39.6)
High-intermediate	158 (43.4)
High	42 (11.5)
B symptom	
Absent	280 (76.9)
Present	84 (23.1)
ECOG PS	
<2	304 (83.5)
≥2	60 (16.5)
LDH	
Normal	158 (43.4)
Abnormal (over 480 IU/L)	206 (56.6)
Bone marrow involvement	
BMB BM involvement	42 (13.0)
PET BM involvement	82 (25.5)
IGR BM involvement	114 (31.3)
FCM BM involvement	47 (12.9)
BMC BM involvement	37 (10.2)
Relapse	123 (33.8)
Death	64 (17.6)

ECOG PS, Eastern Cooperative Oncology Group performance status; LDH, lactate dehydrogenase; PET/CT, positron emission tomography-computed tomography; IGR, immunoglobulin gene rearrangement; FCM, flow cytometry; BMC, bone marrow cytology; BMB, bone marrow biopsy pathology; IPI, International Prognostic Index; NCCN-IPI, National Comprehensive Cancer Network-IPI.

### Higher detection rates of BMI assessed by IGR in DLBCL patients

Our data showed that there existed different detection rates with five kinds of examinations for BMI evaluation. Clonality testing identified IGR BM involvement-positive in 114 patients (31.3%), while 47 (12.9%) patients tested positive for FCM BM involvement. 37 (10.2%) patients were diagnosed with BMC BM involvement. BMB detected positive BMI in 42 cases (13.0%; concordant BMB BM involvement=27 and inconsistent BMB BM involvement=15). PET/CT uptake was observed in 82 patients (25.5%), with focal lesions identified in 55 cases (67.1%) and diffuse lesions in 27 cases (32.9%) (**Table 2**).

**Table 2:** Distribution of all patients with bone marrow assessment using five methods.

	BMB BMI (−)	BMB BMI (+)
PET BMI (−)	264	18
PET BMI (+)	58	24
IGR BMI (−)	239	11
IGR BMI (+)	83	31
FCM BMI (−)	298	19
FCM BMI (+)	24	23
BMC BMI (−)	312	15
BMC BMI (+)	10	27

PET/CT, positron emission tomography-computed tomography; IGR, immunoglobulin gene rearrangement; FCM, flow cytometry; BMC, bone marrow cytology; BMB, bone marrow biopsy pathology; BMI, bone marrow involvement.

In the IGR BM involvement results, there were 31 cases (27.2%) showing consistency between IGR BM involvement and BMB BM involvement. However, there were 83 (72.8%) patients whose IGR BM involvement did not match their BMB BM involvement. It is worth noting that only 11 (26.2%) of the BMB BM involvement-positive patients had negative IGR BM involvement results. The number of FCM BM involvement positive patients was 47, while BMC BM involvement positive patients were 37. Among them, 23 (54.8%) and 27 (64.3%) patients in each group were detected with BM involvement by BMB (**Table 2**). The detection rate of IGR BM involvement positive is also the highest among stages IV patients. The detained information of five kinds of detection for BMI in stages IV patients is shown in **Supplementary Table 1**.

### High sensitivity and negative predictive value of IGR for BMI in the patients with DLBCL

Based on the simple method, all four examination modalities demonstrated high negative predictive values in all patients, with FCM and BMC exhibiting relatively high specificity of 92.5% and 96.9%, respectively (shown in **Table 3.1**). Among stage IV patients, IGR has a higher negative predictive value (89.4%) compared to the other three methods, while FCM and BMC exhibit high specificity of 90.0% and 92.3%, respectively. Among the inspection methods involving pairwise collaboration, combining IGR with BMC had highest sensitivity (88.1%; 88.1%) and negative predictive value (97.9%; 94.7%), while combining FCM with BMC had highest specificity (98.4%; 96.2%) for all patients and stage IV patients, respectively (shown in **Table 3.2**). These findings suggest that IGR offers high sensitivity and a strong negative predictive value for BMI assessment.

**Table 3.1:** Assessment of bone marrow involvement for all patients by various examination methods.

Variables	Sensitivity	Specificity	PPV	NPV	95%CI^C^
PET/CT^a^	57.1	82.0	29.3	93.6	0.901-0.959
IGR^a^	73.8	74.2	27.2	95.6	0.923-0.975
FCM^a^	54.8	92.5	48.9	94.0	0.908-0.961
BMC^a^	64.3	96.9	73.0	95.4	0.926-0.972
PET/CT or IGR^a, b^	85.7	63.4	23.4	97.1	0.939-0.987
PET/CT and IGR^a, b^	45.2	92.9	45.2	92.9	0.895-0.952
PET/CT or FCM^a, b^	71.4	78.3	30.0	95.5	0.922-0.974
PET/CT and FCM^a, b^	40.5	96.3	58.6	92.5	0.892-0.949
PET/CT or BMC^a, b^	78.6	80.7	34.7	96.7	0.938-0.982
PET/CT and BMC^a, b^	42.9	98.1	75.0	92.9	0.897-0.952
IGR or FCM^a, b^	83.3	72.4	28.2	97.1	0.941-0.986
IGR and FCM^a, b^	45.2	94.4	51.4	93.0	0.897-0.953
IGR or BMC^a, b^	88.1	73.0	29.8	97.9	0.952-0.991
IGR and BMC^a, b^	50.0	98.1	77.8	93.8	0.907-0.959
FCM or BMC^a, b^	73.8	91.0	51.7	96.4	0.936-0.980
FCM and BMC^a, b^	45.2	98.4	79.2	93.2	0.901-0.955

a Values shown in **Table 2** were used for calculations as standard formulas for sensitivity, specificity, PPV, NPV.

b Parallel test was used to determine sensitivity, specificity of IGH or PET/CT. Serial test was used to determine sensitivity and specificity of combined IGR and PET/CT for detecting BMI.

^C^95% CI for NPV were shown in **Table 3.1**.

**Table 3.2:** Assessment of bone marrow involvement for stage IV patients by various examination methods.

Variables	Sensitivity	Specificity	PPV	NPV	95%CI^C^
PET/CT^a^	57.1	60.0	31.6	81.2	0.723-0.878
IGR^a^	73.8	71.5	45.6	89.4	0.820-0.940
FCM^a^	54.8	90.0	63.9	86.0	0.792-0.909
BMC^a^	64.3	92.3	73.0	88.9	0.825-0.931
PET/CT or IGR^a, b^	85.7	46.9	34.3	91.0	0.818-0.958
PET/CT and IGR^a, b^	45.2	84.6	48.7	82.7	0.754-0.882
PET/CT or FCM^a, b^	71.4	56.9	34.9	86.0	0.772-0.918
PET/CT and FCM^a, b^	40.5	93.1	65.4	82.9	0.759-0.881
PET/CT or BMC^a, b^	78.6	56.9	37.1	89.2	0.807-0.942
PET/CT and BMC^a, b^	42.9	95.4	75.0	83.8	0.770-0.889
IGR or FCM^a, b^	83.3	69.2	46.7	92.8	0.858-0.965
IGR and FCM^a, b^	45.2	92.3	65.5	83.9	0.770-0.890
IGR or BMC^a, b^	88.1	68.5	47.4	94.7	0.881-0.977
IGR and BMC^a, b^	50.0	95.4	77.8	85.5	0.789-0.903
FCM or BMC^a, b^	73.8	86.2	63.3	91.1	0.847-0.949
FCM and BMC^a, b^	45.2	96.2	79.2	84.5	0.778-0.894

a Values shown in **Supplementary Table 1** were used for calculations as standard formulas for sensitivity, specificity, PPV, NPV.

b Parallel test was used to determine sensitivity, specificity of IGH or PET/CT. Serial test was used to determine sensitivity and specificity of combined IGR and PET/CT for detecting BMI.

^C^95% CI for NPV were shown in **Table 3.2**.

### Clinical correlation analysis based on the different BMI examinations

Positive PET BM involvement was clinically associated with different clinical stages (P<0.001) and IPI risk groups (P<0.001), while positive IGR BM involvement rates was significantly associated with different clinical stages (P<0.001), IPI (P<0.001) and NCCN-IPI (P=0.002). Additionally, positive correlations were also found between FCM BM involvement status and clinical stages (P<0.001), IPI (P=0.001), and NCCN-IPI (P=0.010), between BMC BM involvement or BMB BM involvement and different clinical stages (P<0.001), IPI (P<0.001), and NCCN-IPI (**Table 4**).

**Table 4:** Clinical characteristics of all patients with DLBCL according to the five methods (n=364).

Characteristics	NO.	PET BMI (%)	*P* value	IGH BMI (%)	*P* value	FCM BMI (%)	*P* value	BMC BMI (%)	*P* value	BMB BMI (%)	*P* value
Stage											
I	73	0 (0%)	<0.001 (StageI-IIvs. III-IV)	13 (17.8%)	<0.001	1 (1.4%)	<0.001	0 (0%)	<0.001	0 (0%)	<0.001
II	69	0 (0%)		14 (20.3%)		4 (5.8%)		0 (0%)		0 (0%)	
III	50	6 (12.0%)		19 (38.0%)		6 (12.0%)		0 (0%)		0 (0%)	
IV	172	76 (44.2%)		68 (39.5%)		36 (20.9%)		37 (21.5%)		42 (24.4%)	
IPI score											
Low	106	0 (0%)	<0.001 (IPI low vs. High)	17 (16.0%)	<0.001	5 (4.7%)	0.001	0 (0%)	<0.001	0 (0%)	<0.001
Low-intermediate	64	15 (23.4%)		19 (29.7%)		6 (9.4%)		2 (3.1%)		3 (4.7%)	
High-intermediate	104	33 (31.7%)		35 (33.7%)		16 (15.4%)		12 (11.5%)		17 (16.3%)	
High	90	34 (37.8%)		43 (47.8%)		20 (22.2%)		23 (25.6%)		22 (24.4%)	
NCCN IPI score											
Low	20	0 (0%)	0.32 (NCCN IPI low vs. high)	3 (15.0%)	0.002	1 (5.0%)	0.01	0 (0%)	<0.001	0 (0%)	<0.001
Low-intermediate	144	33 (22.9%)		35 (24.3%)		12 (8.3%)		6 (4.2%)		4 (2.8%)	
High-intermediate	158	36 (32.8%)		50 (31.6%)		20 (12.7%)		18 (11.4%)		24 (15.2%)	
High	42	13 (31.0%)		26 (61.9%)		14 (33.3%)		13 (31.0%)		14 (33.3%)	

### Poor survival outcomes of positive BMI by any BMI examinations in all cases with DLBCL

**Figure 1:**
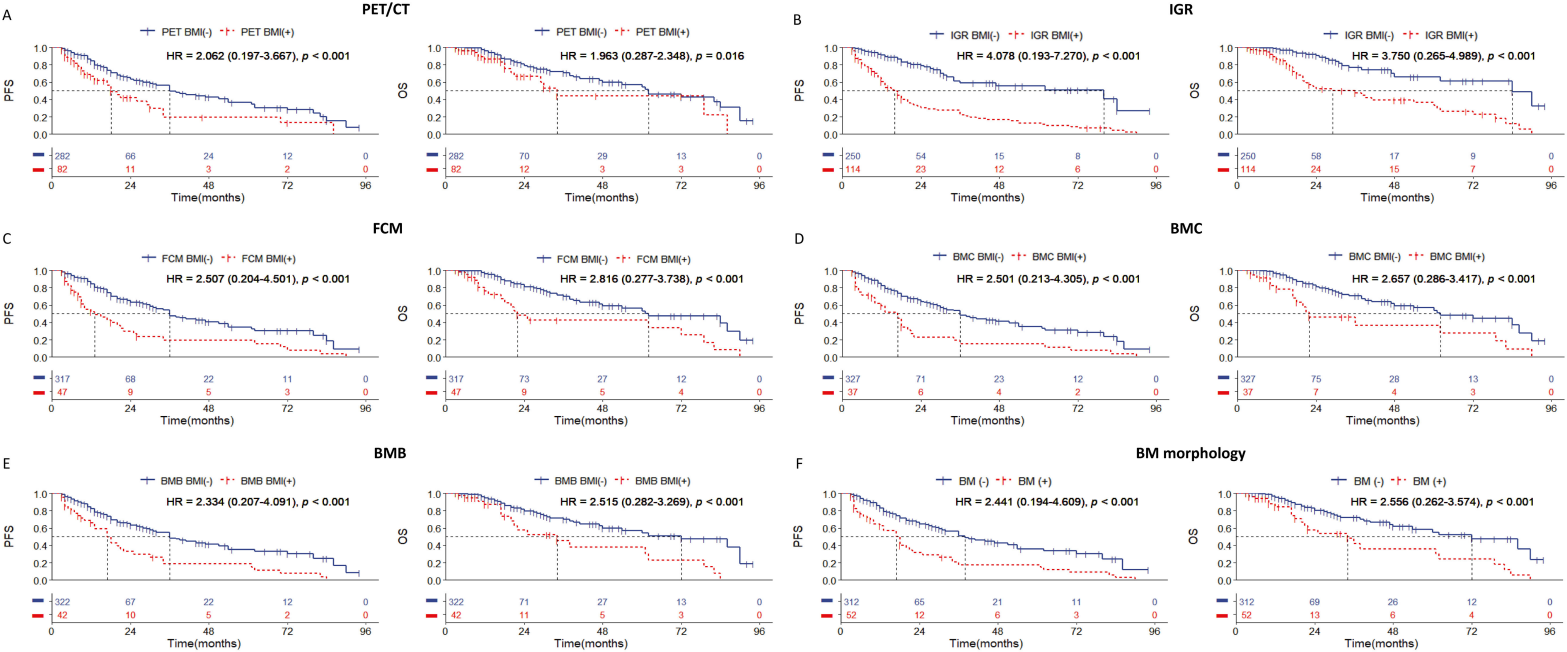
Kaplan-Meier survival curves of all patients with DLBCL according to the BM involvement (BMI) assessed by **(A)** PET/CT; **(B)** immunoglobulin gene rearrangement; **(C)** flow cytometry; **(D)** bone marrow cytology; **(E)** bone marrow biopsy pathology; **(F)** bone marrow morphology. Survival panels present the PFS curves (left) and OS curves (right). Statistical differences were calculated using the log rank test.

In this study, the median follow-up time was 48.5 months (range: 3 to 94 months). During the follow-up period, 123 patients (33.8%) experienced disease progression, and 64 patients (17.6%) died. Evaluation of BMI in all DLBCL cases showed that the patients who tested positive by any examination method had worse PFS and OS (**Figure 1**). In this cohort, although there is a statistically significant difference in prognosis among PET/CT whose HR value is the lowest (**Figure 1A**), and IGR was a better indicator of survival prognosis of PFS and OS with the highest HR value (**Figure 1B**), and the ROC curve results also showed that IGR had the highest predictive efficiency, while PET CT had the lowest predictive efficiency (**Supplementary Figure 1**). In addition, we found that morphology can be better of survival prognosis when divided into BMC and BMB (**Figures 1D–F**).

### Significant discrimination of PFS and OS between IGR BM involvement positive and negative cases with DLBCL of stages I to III

**Figure 2:**
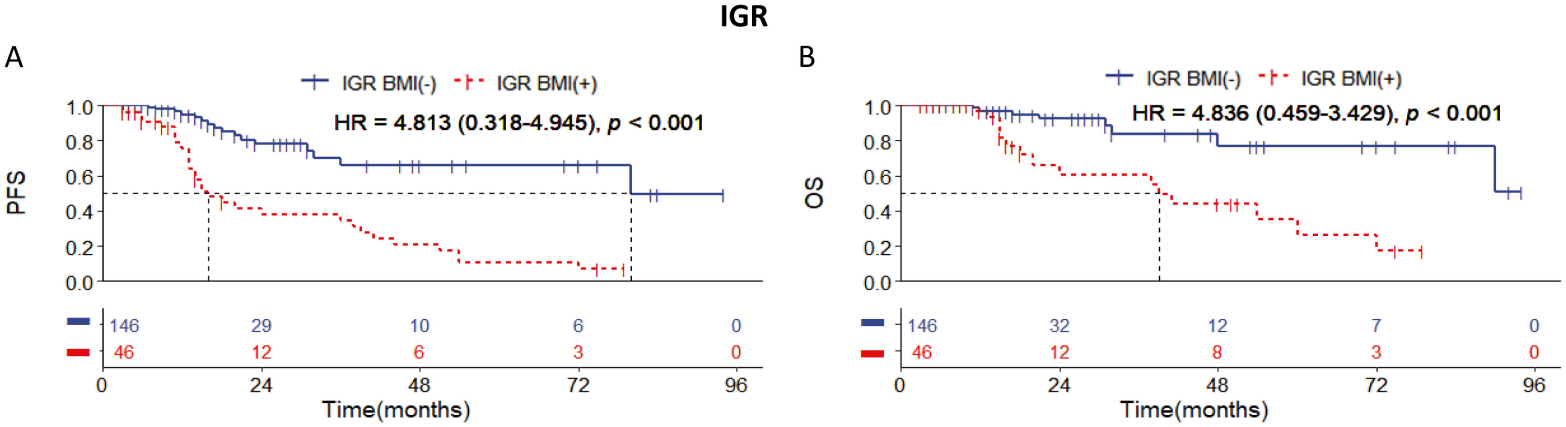
Kaplan-Meier survival curves of patients of stage I-III with DLBCL according to IGR BM involvement. Survival panels present **(A)** the PFS curves and **(B)** the OS curves. Statistical differences were calculated using the log rank test.

Among the 192 patients with stages I to III, our data revealed that IGR BM involvement-positivity was observed in 46 cases, significantly more than other methods. Kaplan-Meier (KM) survival analysis showed that cases with IGR BM involvement-positive had worse PFS and OS compared to IGR BM involvement-negative cases. Of these 46 cases, 30 experienced relapse or death during long-term follow-up (**Figure 2**). These findings suggest that IGR may be more effective at detecting small lesions in BMI compared to other examination methods.

### Poorer survival outcomes in the positive of IGR BM involvement in the DLBCL cases with stages IV

**Figure 3:**
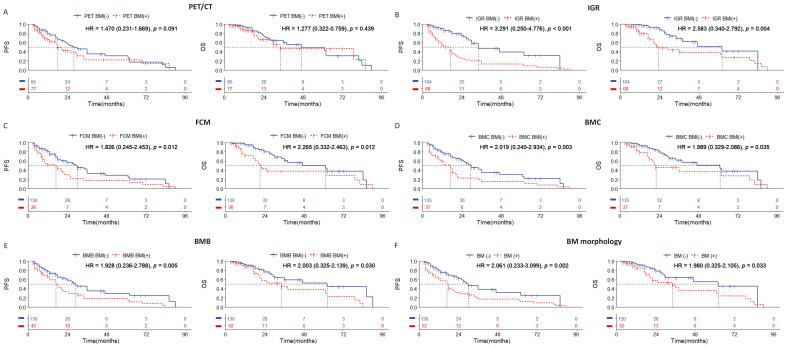
Kaplan-Meier survival curves of patients of stage IV with DLBCL according to the BMI assessed by **(A)** PET/CT; **(B)** immunoglobulin gene rearrangement; **(C)** flow cytometry; **(D)** bone marrow cytology; **(E)** bone marrow biopsy pathology; **(F)** bone marrow morphology. Survival panels present the PFS curves (left) and OS curves (right). Statistical differences were calculated using the log rank test.

Among the 172 patients with stage IV DLBCL in this cohort, we observed statistically significant differences in survival prognosis between patients who were BMI-negative and BMI-positive, as determined by IGR, FCM, BMC, and BMB examination methods (**Figure 3**). Notably, the distinction in PFS and OS was most pronounced between IGR BM involvement-positive and BMI-negative cases, with IGR-positive cases showing a higher hazard ratio (**Figure 3B**). In contrast, no significant differences in survival prognosis were found between PET BM involvement-positive and BM involvement-negative cases (**Figure 3A**). These results indicate that IGR assessment provides a significant advantage in detecting BMI for stage IV DLBCL patients, while PET/CT may have limitations in this specific group.

### Combining IGR and BMB is the most effective way to predict prognosis in the DLBCL patients with stage IV

**Figure 4:**
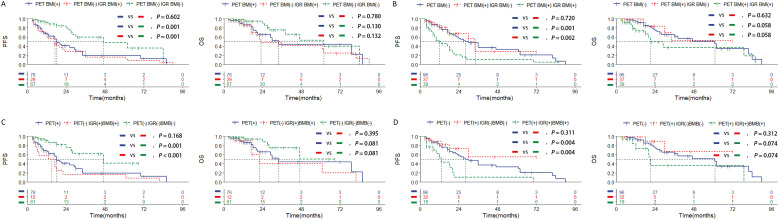
Kaplan-Meier survival curves of patients of stage IV with DLBCL in training set according to the BMI assessed by **(A, B)** combined assessment with PET/CT and immunoglobulin gene rearrangement; **(C, D)** combined assessment with PET/CT, immunoglobulin gene rearrangement and bone marrow biopsy pathology. Survival panels present the PFS curves (left) and OS curves (right). Statistical differences were calculated using the log rank test.

Given the lack of statistical significance in survival prognosis among stage IV patients with PET BM involvement, our study will concentrate on stage IV patients assessed by PET/CT and conduct KM curve analysis to explore which examination method can address these limitations. The survival rate of patients with negative PET BM involvement but positive IGR BM involvement (n=29) was similar to that of patients with positive PET BM involvement (n=76), and statistically different from that of negative PET BM involvement and negative IGR BM involvement patients (n=67) (**Figure 4A**). On the other hand, the survival rate of patients with PET BM involvement (+) and IGR BM involvement (-) (n=37) was similar to that of patients with PET BM involvement (-), and statistically different from that of PET BM involvement (+) and IGR BM involvement (+) patients (**Figure 4B**). The combination of PET/CT with other single examination methods is listed in the **Supplementary Figure 2**. The above results indicate that the PET/CT examination for detecting BMI does have limitations, which may result in false negatives and false positives. Among the single examination methods, IGR demonstrates the strongest ability to further differentiate prognosis among the patients with PET/CT (**Supplementary Table 3**).

Among the various inspection methods involving pairwise combinations, survival analysis revealed that patients who tested double-positive exhibited a worse prognosis than single-positive patients, and significantly worse than double-negative patients (**Supplementary Figure 3**; **Supplementary Table 3**). Moreover, the combination of IGR BM involvement (+), and BMB BM involvement (+) was significantly associated with shorter survival outcomes than other combinations in the patients with PET BM involvement (-) (**Figure 4C**; **Supplementary Table 3**). Additionally, patients with negative IGR BM involvement and negative BMB BM involvement were significantly associated with longer survival outcomes than other combinations in the patients with positive PET BM involvement (**Figure 4D**; **Supplementary Table 3**). Other pairwise collaborations are shown in **Supplementary Figure 4** and **Supplementary Figure 5**. The ROC curve results further show that the combination of IGR and BMB has better predictive efficacy than other methods, and even better than IGR and BMB (**Supplementary Figure 6**). These findings suggest that PET/CT assessment may result in false negatives and false positives in stage IV DLBCL cases and combining IGR with BMB is the most effective way, even better than IGR, to overcome this limitation and predict prognosis more effectively.

### The double-positive of IGR BM involvement and BMB BM involvement at diagnosis as a poor prognostic factors

To investigate the association between IGR BM involvement and BMB BM involvement with clinical outcomes, we performed the Cox proportional hazards analysis. We divided all patients (n=364) into a training set (n=243) and a testing set (n=121) in a 2:1 ratio. No statistically significant differences were found between the clinical data of the two sets (**Supplementary Table 4**). In the training set, we evaluated the relationship between clinical parameters and PFS, OS. Univariate analysis revealed that Age (≥60, P<0.001), B symptom (P=0.009), Eastern Cooperative Oncology Group (ECOG) performance score (≥2, P=0.039), IPI (≥3, P=0.001), NCCN-IPI (≥3, P<0.001), and the combination of any two examination methods (P<0.001) were associated with PFS. Factors with P ≤ 0.001 were then included in the multivariate analysis. Multivariate Cox-regression analysis showed that the combined assessment of IGR BM involvement and BMB BM involvement (P=0.026; HR, 2.054; 95%CI, 0.914-4.616), NCCN-IPI (≥3; P=0.002; HR, 1.811; 95%CI, 1.237-2.652), and Age (≥60; P=0.035; HR, 1.651; 95%CI, 0.853-3.057) were independent prognostic factors of PFS. Additionally, Age (≥60, P<0.001), NCCN-IPI (≥3, P=0.001), combined assessment of IGR BM involvement and FCM BM involvement (P<0.001), combined assessment of IGR BM involvement and BMC BM involvement (P<0.001), and combined assessment of IGR BM involvement and BMB BM involvement (P<0.001) were associated with OS. Multivariate Cox-regression analysis showed that double-positive IGR BM involvement and BMB BM involvement (P=0.042; HR, 1.670; 95%CI, 0.656-4.250) was an independent prognostic factors of OS (**Table 5**).

**Table 5:** Univariate and multivariate Cox-proportional hazard regression analyses predicting PFS and OS in DLBCL patients.

Variables	PFS				OS		
	Univariate *P* value	Multivariate HR (95% CI)	*P* value	Score	Univariate *P* value	Multivariate HR (95% CI)	*P* value
Age (≥60)	<0.001	1.651(0.853-3.057)	0.035	1	<0.001	1.692(0.685-4.180)	0.054
B symptom	0.009				0.534		
ECOG PS (≥2)	0.039				0.186		
Stage (≥3)	0.079				0.041		
LDH (>normal)	0.046				0.014		
IPI (≥3)	0.001	1.034(0.823-1.299)	0.773		0.002		
NCCN-IPI (≥3)	<0.001	1.811(1.237-2.652)	0.002	1	0.001	1.502(0.840-2.687)	0.170
Combined IGR and FCM	<0.001	1.548(0.725-3.309)	0.259		<0.001	1.008(0.363-2.798)	0.382
Combined IGR and BMC	<0.001	0.835(0.436-1.597)	0.082		<0.001	1.557(0.460-5.263)	0.076
Combined IGR and BMB	<0.001	2.054(0.914-4.616)	0.026	2	<0.001	1.670(0.656-4.250)	0.042
Combined FCM and BMC	<0.001	0.616(0.394-0.963)	0.585		0.002		
Combined FCM and BMB	<0.001		\^a^		0.002		
Combined BMC and BMB	<0.001		\^a^		0.001	0.703(0.363-1.361)	0.295

Univariate analysis and multivariate Cox-proportional hazard regression analysis was conducted using the Breslow method. Multivariate analysis was performed using the covariates, which showed a P value of less than 0.001 in the univariate analysis.

DLBCL, diffuse large B-cell lymphoma; CI, confidence interval; HR, hazard ratio; ECOG PS, Eastern Cooperative Oncology Group performance score; LDH, lactate dehydrogenase.

a Decreases in degrees of freedom due to constant or linear dependent covariates.

### Development of a modified risk stratification model of IPI and NCCN-IPI by adding IGR BM involvement and BMB BM involvement

**Figure 5:**
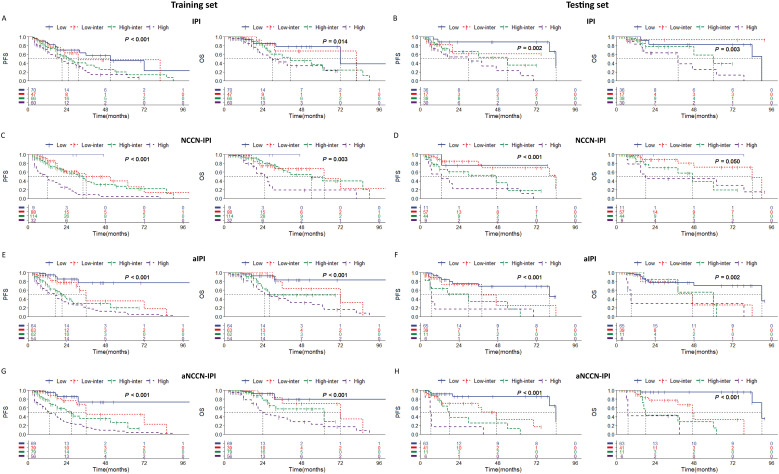
Kaplan-Meier survival curves of patients (1) in training set according to **(A)** IPI; **(C)** NCCN-IPI; **(E)** adjusted IPI; **(G)** adjusted NCCN-IPI. (2) in testing set according to **(B)** IPI; **(D)** NCCN-IPI; **(F)** adjusted IPI; **(H)** adjusted NCCN-IPI. Survival panels present the PFS curves (left) and OS curves (right). Statistical differences were calculated using the log rank test. High = rated as high risk, High inter = rated as high-intermediate risk, Low inter = rated as low-intermediate risk, Low = rated as low risk.

Multivariate analysis identified the combination of IGR BM involvement, BMB BM involvement as an independent prognostic factors in the training set. Based on these result, we aimed to develop a new prognostic model for the DLBCL and have made the following regulations. The presence of both IGR BM involvement (+) and BMB BM involvement (+) contributes two points, while the presence of either IGR BM involvement (+) or BMB BM involvement (+), Age≥60, or NCCN-IPI≥3 contributes one point each. The presence of both IGR BM involvement (-) and BMB BM involvement (-) contributes zero points. Risk categories were classified as follows: Low risk (0 points), Low-intermediate risk (1 point), High-intermediate risk (2 points), and High risk (3-4 points). In the new adjusted IPI, an IPI score of≥3 is assigned one point, while the other criteria remain consistent with the adjusted NCCN-IPI. Both in the training and testing sets, the adjusted risk stratification model demonstrates a more pronounced difference in patient survival outcomes across various risk categories (**Figure 5**).

**Figure 6:**
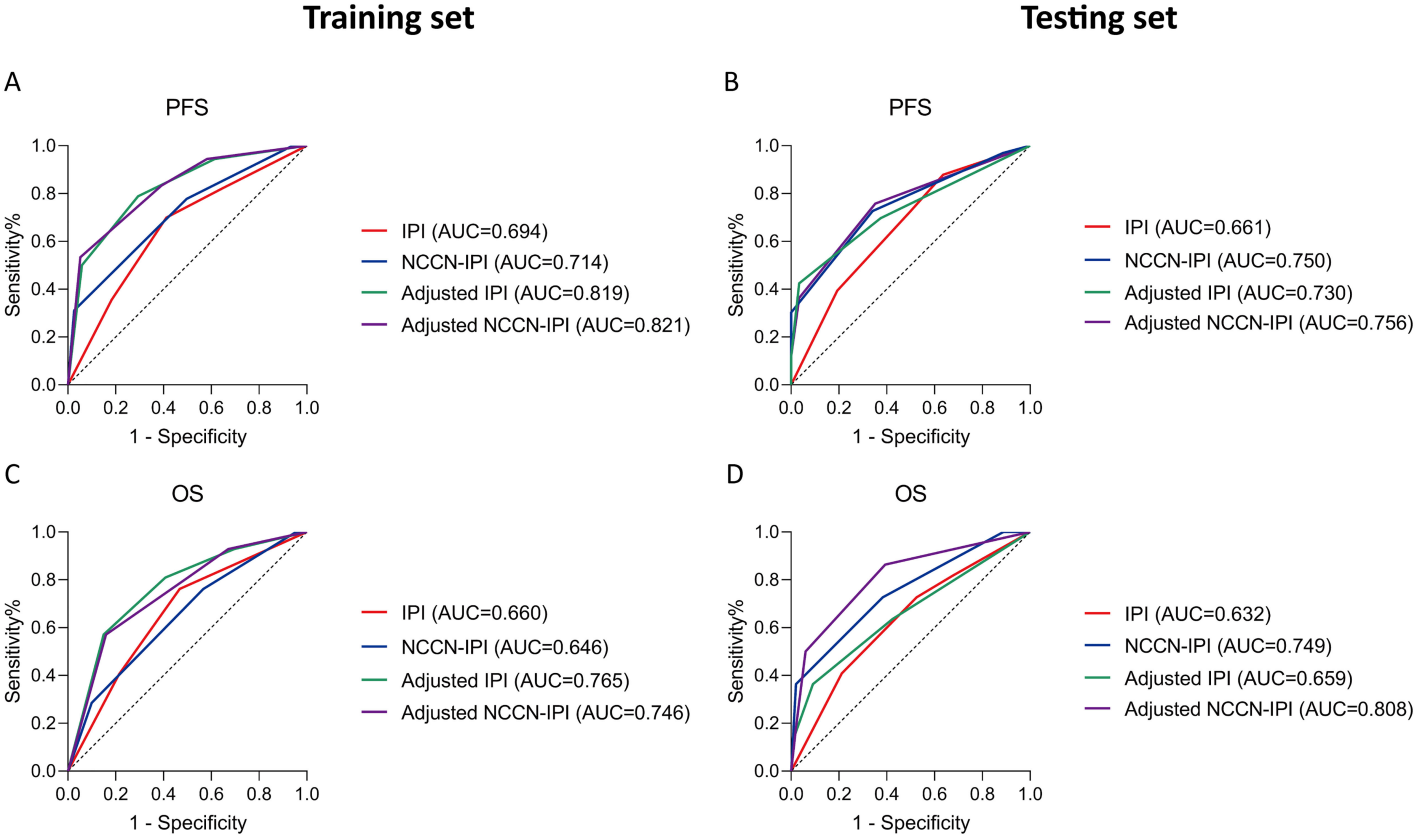
Receiver operating characteristic (ROC) curves of all patients according to the survival status assessed in (**A, C**) training set; (**B, D**) testing set. Survival panels present the PFS curves (left) and OS curves (right). The Area under Curve (AUC) were calculated using the Mann-Whitney U test.

Additionally, the ROC curve indicates that the adjusted NCCN-IPI outperforms the original NCCN-IPI in evaluation performance (**Figure 6**). Furthermore, the adjusted IPI also outperforms the original IPI in evaluation performance in both the training and testing sets (**Figure 6**). Across all patients, the new model also demonstrated more accurate survival predictions (**Supplementary Figures 7, 8**). Moreover, the C-index of the scoring system improved across all three cohorts after adjustment (**Table 6**).

**Table 6:** Harrell’s C statistic for discriminatory values on survival.

Parameter	Training set	Testing set	All patients
IPI	0.687 (0.555-0.818)	0.700 (0.569-0.831)	0.629 (0.549-0.709)
NCCN-IPI	0.725 (0.566-0.885)	0.789 (0.681-0.898)	0.752 (0.678-0.826)
Adjusted IPI	0.781 (0.678-0.883)	0.782 (0.682-0.882)	0.767 (0.708-0.825)
Adjusted NCCN-IPI	0.815 (0.728-0.902)	0.776 (0.659-0.894)	0.814 (0.761-0.868)

## Discussion

Infiltration of lymphoma cells into the bone marrow is a poor prognostic factor in DLBCL (1). Studies have demonstrated that even minute lesions involving the bone marrow can significantly impact patients’ prognosis (22–24). Clinically, occult bone marrow infiltration is a challenge to detect using PET/CT or BMB due to methodological limitations. We compared five commonly used clinical methods of detection methods, including IGR. Our data indicated that IGR is more effective in detecting BMI than others, which often fails to identify the hidden presence of infiltration. Based on these findings, we established a novel scoring system which demonstrates a better ability to stratify prognosis. To our knowledge, this is the first multi-center study to analyze imaging, molecular, morphology, and flow immunophenotype data to explore BMI and its correlation with survival outcomes. Notably, we first found that IGR-based clonality detection enhances the definition of BMI in DLBCL and may potentially replace PET/CT for this purpose in the future.

When diagnosing BMI in all patients, the agreement between BMC and BMB, both commonly considered morphological examinations, is only moderate. Diagnosis based on morphological and cytomorphological features may be subjective and vary among different subsets of bone marrow invasion (1). This variability underscores the need to categorize morphology into BMC and BMB. Among the 42 patients with positive BMB results, there were only 11 patients (26.2%) exhibited negative IGR BM involvement, which was less than other methods. In these 11 patients, FCM analysis revealed low-level involvement in 4 cases, with abnormal cell proportions of 14.3%, 6.6%, 1.8%, and 0.3%, respectively. Additionally, three instances of recurrence were observed among these patients, demonstrating the utility of FCM as an auxiliary method for detecting minimal residual disease (MRD). Studies have demonstrated that neither morphology nor flow cytometry alone is sufficient for detecting of all cases of NHL with BMI. However, flow cytometry exhibits higher sensitivity in detecting and encompassing occult bone marrow infiltration (25, 26), while morphology is effective for identifying most cases with involvement exceeding 5% (27). Therefore, compared to PET/CT, IGR and FCM, due to their high sensitivity, can complement morphology to provide a more comprehensive diagnosis of BMI.

In cases of NHL, DLBCL is particularly prone to occult bone marrow involvement, with lesions typically presenting as very small clonal cell populations that account for only 0.09% to 3% of the total cells analyzed (28). However, in the clinical diagnostic process, small bone marrow lesions and low sensitivity in morphological examination often lead to misclassification of patients with bone marrow involvement as non-stage IV. Among the 192 patients in stage I-III, IGR has proven to be a relatively sensitive auxiliary inspection method and is associated with poor prognosis. This finding aligns with the results reported by Hohendanner et al. and Cho et al (29, 30). Hohendanner et al. argue that the use of molecular methods enhances the precision in staging DLBCL patients and identifies a subset of patients with histologically normal bone marrow, but with significantly poorer overall survival due to molecular detection of bone marrow involvement^29^. Our study demonstrates that IGR has good prognostic predictive ability in stage I-III patients, indicating its potential for sensitively detecting ‘hidden bone marrow infiltrations’ and guiding clinical staging. This highlights the critical importance of using the highly sensitive IGR method for diagnosing BMI in DLBCL. There are currently two common methods for gene rearrangement: BIOMED2 PCR and Next-generation Sequencing (NSG). BIOMED2 PCR is based on low cost and rapidity, and is suitable for routine screening and resource-limited scenarios (20, 31), while NGS is known for its high sensitivity and multi-dimensional analysis (32, 33). It is a core tool for the diagnosis and dynamic monitoring of complex cases in the era of precision medicine. In this study, gene rearrangement was detected by BIOMED2 PCR. The main reason is that the patient population in large centers is complex and more suitable for economical detection methods. Although the NSG detection method is accurate, it takes a long time and is not suitable for patients with more severe clinical manifestations. In the future, BIOMED2 PCR can be used as an initial screening to quickly exclude polyclonal lesions and reduce the burden of NGS testing. NGS is used for in-depth analysis and further sequencing of PCR-positive samples to obtain molecular details. The two will complement each other and jointly promote the molecular stratification and personalized treatment of hematological tumors.

The comparison between PET/CT and BMB for the identification of BMI has been widely discussed, but there is no consensus on the utilization of PET/CT or BMB for assessing BMI (5, 6). In this study, PET/CT exhibited moderate sensitivity (57.1%) and specificity (82.0%), consistent with findings by Thanarajasingam et al (3). Among stage IV patients, there was no statistically significant difference in PFS and OS between the two groups of patients with PET/CT. Notably, 40 out of the 96 patients with negative PET/CT BM involvement results experienced relapse, suggesting that PET/CT may lack sufficient sensitivity for detecting BMI. This finding contradicts the recommendation by Adams et al. to routinely use PET/CT in DLBCL across most centers (34, 35). Elstrom et al. defined a positive ^18^FDG-PET/CT scan as having a specific SUV greater than 2.5 (36). Although this binary classification is convenient, it has significant limitations due to variations in patients’ basal metabolic activity. Therefore, we believe that the current approach of categorizing PET/CT results as simply positive or negative may overlook critical clinical details, leading to inaccurate outcomes.

When analyzing the relationship between diffuse PET/CT uptake and BMB BM involvement, we find 55 cases with localized PET/CT uptake, of which 46 were associated with negative BMB BM involvement. Among the 27 cases with diffuse PET/CT uptake, 17 patients had positive BMB BM involvement (**Supplementary Table 2**). Patients with diffuse uptake generally had poor prognosis, consistent with the findings of Hong et al. (7) and Adams et al (37). However, there is no current consensus on interpreting diffuse bone marrow uptake on FDG PET as definitive evidence of bone marrow involvement (38), highlighting the need for further research in this area. Overall, PET/CT plays an indispensable role in assessing the impact of tumors on various tissues and organs by evaluating metabolic activity from a comprehensive perspective (39). Nevertheless, significant limitations remain in determining bone marrow infiltration. Given the advancements in auxiliary examination methods, such as molecular detection, it is questionable whether PET/CT alone can reliably predict prognosis (40).

In multivariate regression analysis, combining IGR with BMB resulted in the highest hazard ratio (HR) value, and patients with the double-positive results had the poorest prognosis. This outcome aligns with the findings of Cho et al., who indicated that monoclonal and histological B-cell accumulation in the bone marrow is strongly associated with poor prognosis and can effectively identify high-risk DLBCL patients (30). The discrepancies between the two methods might stem from technical factors, raising concerns about their clinical relevance. Although IGH can directly diagnose BMI in lymphoma by detecting B-cell clones, previous research have indicated the necessity of incorporating “dominant tissue-matched clonotype” due to the presence of distinct molecular subgroups of B-cells in DLBCL (41, 42). While BM smear analysis remains essential in lymphoma management, as it can detect other hematological abnormalities such as myelodysplasia and hemophagocytosis, the presence of BMB BM involvement (+) in patients was closely associated with clinical parameters such as abnormal LDH, B symptoms, IPI≥3, and NCCN-IPI≥3 in this study. Therefore, we advocate for the judicious use of both IGR and BMB in clinical practice. This approach can avoid the limitations of PET/CT in detecting BMI and provide more comprehensive diagnostic information, thereby enhancing the prognostic value for patients.

Given the significance of BMI as assessed by IGR and BMB, we developed a novel adjusted risk stratification model in this study. By integrating IGR and BMB with IPI and NCCN-IPI, the adjusted model demonstrated superior performance compared to the original model, as indicated by ROC curve analysis. Specifically, the adjusted IPI showed marked improvement over the original IPI. However, for NCCN-IPI, none of the 9 patients in the low-risk group experienced relapse in the training set, confirming its effectiveness in identifying low-risk individuals. In contrast, the adjusted NCCN-IPI more accurately identified patients in the intermediate-low-risk and intermediate-high-risk categories. Consistent with our findings, Ruppert et al. also suggested that NCCN-IPI refines the IPI by better identifying high-risk groups with less heterogeneity. Unlike the IPI, which fails to account for occult or morphologically inconspicuous bone marrow involvement (43), the adjusted model achieves more accurate risk stratification by incorporating IGR.

This study also has several limitations. Firstly, the influence of different examination methods on treatment strategies was not analyzed. Although it was mentioned that all patients received R-CHOP-based chemotherapy, the study did not account for the molecular differences between DLBCL subgroups, which could affect treatment efficacy (44). Secondly, the interpretation of PET/CT results was based solely on a binary system, without specifying clear cutoff values. which may lead to subjective judgments and potential misdiagnosis. Third, the study excluded patients who lacked one or more examination results, which imposes certain limitations on the retrospective analysis. Lastly, as a retrospective analysis, this study requires prospective, independent research, which we will continue to conduct in the future.

In conclusions, our study utilized multi-center data and incorporated a variety of commonly used blood system examination methods. The IGR data demonstrated strong diagnostic capabilities for detecting BMI and predicting patient survival. Building on these results, we developed a novel risk stratification model by integrating IGR and BMB, which includes additional parameters and has shown superior predictive ability for survival compared to traditional models such as IPI and NCCN-IPI. Our findings suggest that IGR is essential for evaluating BMI and serves as an ideal marker for disease staging and risk stratification in DLBCL patients in the rituximab era.

## Data Availability

The raw data supporting the conclusions of this article will be made available by the authors, without undue reservation.
