# Supplementary material for: Development and validation of novel models based on clonality immunoglobulin gene rearrangement for evaluation of bone marrow involvement and prognostic prediction in patients with diffuse large B-cell Lymphoma: a multicenter retrospective study
Source: Front Immunol. 2025 Apr 14;16:1547056. doi: 10.3389/fimmu.2025.1547056 (PMC12034632; doi:10.3389/fimmu.2025.1547056)
Supplement: Supplementary file 1 [file Table1.docx]

**Supplement Table 1.** Distribution of stage Ⅳ patients with bone marrow assessment using five methods.

|  | BMB BMI (−) | BMB BMI (+) |
| --- | --- | --- |
| PET BMI (−)  PET BMI (+) | 78  52 | 18  24 |
| IGR BMI (−)  IGR BMI (+) | 93  37 | 11  31 |
| FCM BMI (−)  FCM BMI (+) | 117  13 | 19  23 |
| BMC BMI (−)  BMC BMI (+) | 120  10 | 15  27 |

PET/CT = positron emission tomography-computed tomography, IGR = immunoglobulin gene rearrangement, FCM = flow cytometry, BMC = bone marrow cytology, BMB= bone marrow biopsy.

**Supplement Table 2.** Uptake status of patients with PET BMI

| PET BMI | BMB BMI (−) | BMB BMI (+) |
| --- | --- | --- |
| Focal FDG BM uptake | 46 | 9 |
| Diffuse FDG BM uptake | 10 | 17 |

PET/CT = positron emission tomography-computed tomography, BMB= bone marrow biopsy.

**Supplement Table 3.** Difference of median survival time in PFS and OS between inspection methods and scoring methods

| Variables | PFS | OS |
| --- | --- | --- |
|  | MST | MST |
| PET/CT(-) | 36^a^; 28^b^ | 62^a^; 61^b^ |
| PET/CT(+) | 18^a^; 28^b^ | 34^a^; 34^b^ |
| IGR(-) | 80^a^; 34^b^ | 84^a^;62^b^ |
| IGR(+) | 16^a^; 17^b^ | 29^a^;23^b^ |
| FCM(-) | 36^a^; 30^b^ | 62^a^; 61^b^ |
| FCM(+) | 17^a^; 13^b^ | 22^a^; 21^b^ |
| BMC(-) | 36^a^; 29^b^ | 62^a^; 61^b^ |
| BMC(+) | 17^a^; 17^b^ | 22^a^; 22^b^ |
| BMB(-) | 36^a^; 29^b^ | 72^a^; 62^b^ |
| BMB(+) | 17^a^; 17^b^ | 34^a^; 34^b^ |
| BM(-) | 38^a^; 30^b^ | 72^a^; 62^b^ |
| BM(+) | 17^a^; 17^b^ | 34^a^; 34^b^ |
| PET/CT(-) IGR(+) | 17 | 23 |
| PET/CT(-) IGR(-) | 47 | 62 |
| PET/CT(-) FCM(+) | 22 | 25 |
| PET/CT(-) FCM(-) | 30 | 61 |
| PET/CT(-) BMC(+) | 18 | 36 |
| PET/CT(-) BMC(-) | 36 | 61 |
| PET/CT(-) BMB(+) | 18 | 38 |
| PET/CT(-) BMB(-) | 36 | 62 |
| PET/CT(+) IGR(-) | 34 | NA |
| PET/CT(+) IGR(+) | 12 | 21 |
| PET/CT(+) FCM(-) | 29 | 34 |
| PET/CT(+) FCM(+) | 10 | 20 |
| PET/CT(+) BMC(-) | 26 | 34 |
| PET/CT(+) BMC(+) | 12 | 20 |
| PET/CT(+) BMB(-) | 26 | 86 |
| PET/CT(+) BMB(+) | 17 | 21 |
| IGR(-) FCM(-) | 47 | 62 |
| IGR(+) or FCM(+) | 17 | 25 |
| IGR(+) FCM(+) | 12 | 21 |
| IGR(-) BMC(-) | 47 | 47 |
| IGR(+) or BMC(+) | 17 | 29 |
| IGR(+) BMC(+) | 13 | 22 |
| IGR(-) BMB(-) | 47 | NA |
| IGR(+) or BMB(+) | 18 | 36 |
| IGR(+) BMB(+) | 13 | 21 |
| FCM(-) BMC(-) | 30 | 61 |
| FCM(+) or BMC(+) | 13 | 36 |
| FCM(+) BMC(+) | 17 | 20 |
| FCM(-) BMB(-) | 30 | 47 |
| FCM(+) or BMB(+) | 18 | 61 |
| FCM(+) BMB(+) | 17 | 21 |
| BMC(-) BMB(-) | 30 | 62 |
| BMC(+) or FCM(+) | 18 | 36 |
| BMC(+) BMB(+) | 17 | 21 |
| PET/CT(-) IGR(+)FCM(+) | 22 | 62 |
| PET/CT(-) IGR(-)FCM(-) | 62 | 62 |
| PET/CT(-) IGR(+)BMC(+) | 18 | 36 |
| PET/CT(-) IGR(-)BMC(-) | 47 | 47 |
| PET/CT(-) IGR(+)BMB(+) | 15 | 21 |
| PET/CT(-) IGR(-)BMB(-) | 47 | 47 |
| PET/CT(-) FCM(+)BMC(+) | 19.5 | 22 |
| PET/CT(-) FCM(-)BMC(-) | 36 | 61 |
| PET/CT(-) FCM(+)BMB(+) | 19.5 | 22 |
| PET/CT(-) FCM(-)BMB(-) | 36 | 47 |
| PET/CT(-) BMC(+)BMB(+) | 17.5 | 22 |
| PET/CT(-) BMC(-)BMB(-) | 36 | 62 |
| PET/CT(+) IGR(-)FCM(-) | 34 | 34 |
| PET/CT(+) IGR(+)FCM(+) | 10 | 20 |
| PET/CT(+) IGR(-)BMC(-) | 34 | NA |
| PET/CT(+) IGR(+)BMC(+) | 12 | 20 |
| PET/CT(+) IGR(-)BMB(-) | NA | NA |
| PET/CT(+) IGR(+)BMB(+) | 13 | 21 |
| PET/CT(+) FCM(-)BMC(-) | 30 | 34 |
| PET/CT(+) FCM(+)BMC(+) | 12 | 20 |
| PET/CT(+) FCM(-)BMB(-) | 30 | 86 |
| PET/CT(+) FCM(+)BMB(+) | 12 | 21 |
| PET/CT(+) BMC(-)BMB(-) | 29 | 86 |
| PET/CT(+) BMC(+)BMB(+) | 13 | 20 |
| IPI |  |  |
| High | 51^c^; 84^d^; 80^a^ | 72^c^; 90^d^; 90^a^ |
| High-intermediate | 31^c^; NA^d^; 31^a^ | 80^c^; NA^d^; 82^a^ |
| Low-inter-mediate | 25^c^; 54^d^; 27^a^ | 41^c^; 60^d^; 54^a^ |
| Low | 21^c^; 30^d^; 24^a^ | 30^c^; 38^d^; 38^a^ |
| NCCN-IPI |  |  |
| High | NA^c^; NA^d^; NA^a^ | NA^c^; NA^d^; NA^a^ |
| High-intermediate | 36^c^; 84^d^; 62^a^ | 62^c^; 84^d^; 84^a^ |
| Low-inter-mediate | 34^c^; 47^d^; 34^a^ | 54^c^; 47^d^; 54^a^ |
| Low | 12^c^; 13^d^; 13^a^ | 25^c^; 18^d^; 27^a^ |
| Adjusted IPI |  |  |
| High | NA^c^; 80^d^; NA^a^ | NAc; 90^d^; NA^a^ |
| High-intermediate | 36^c^; 38^d^; 36^a^ | 72^c^; 47^d^; 72^a^ |
| Low-inter-mediate | 21^c^; 30^d^; 23^a^ | 28^c^; 60^d^; 60^a^ |
| Low | 17^c^; 7^d^; 17^a^ | 25^c^; 9^d^; 25^a^ |
| Adjusted NCCN-IPI |  |  |
| High | NA^c^; 84^d^; 84^a^ | NA^c^; 84^d^; 90^a^ |
| High-intermediate | 36^c^; 47^d^; 38^a^ | 72^c^; 47^d^; 72^a^ |
| Low-inter-mediate | 27^c^; 18^d^; 24^a^ | 62^c^; 18^d^; 60^a^ |
| Low | 13^c^; 6.5^d^; 12^a^ | 24^c^; 7^d^; 22^a^ |

PET/CT = positron emission tomography-computed tomography, IGR = immunoglobulin gene rearrangement, FCM = flow cytometry, BMC = bone marrow cytology, BMB= bone marrow biopsy, PFS = progression-free survival, OS = overall survival, IPI = International Prognostic Index, NCCN-IPI = National Comprehensive Cancer Network-IPI.

^a^Statistics in all patients.

^b^Statistics in patients of stage Ⅳ

^c^Statistics in training set

^d^Statistics in testing set

**Supplement Table 4.** The comparison of baseline clinical characteristics between Tranting set and Testing set

| Characteristics | Training set | Testing set | P value |
| --- | --- | --- | --- |
| Sex (male) | 129 | 61 | 0.631 |
| Age ( ≥ 60) | 134 | 62 | 0.481 |
| B symptom | 56 | 28 | 0.984 |
| ECOG PS ( ≥ 2) | 46 | 14 | 0.075 |
| Stage ( ≥ 3) | 155 | 67 | 0.121 |
| LDH ( > normal) | 156 | 50 | 0.221 |
| IPI ( ≥ 3) | 126 | 68 | 0.434 |
| NCCN-IPI ( ≥ 3) | 141 | 54 | 0.485 |
| BMI | 117 | 49 | 0.167 |
| Relapse | 90 | 33 | 0.064 |
| Death | 42 | 22 | 0.832 |
