# Supplementary material for: Development and validation of novel models based on clonality immunoglobulin gene rearrangement for evaluation of bone marrow involvement and prognostic prediction in patients with diffuse large B-cell Lymphoma: a multicenter retrospective study
Source: Front Immunol. 2025 Apr 14;16:1547056. doi: 10.3389/fimmu.2025.1547056 (PMC12034632; doi:10.3389/fimmu.2025.1547056)
Supplement: Supplementary file 2 [file DataSheet1.docx]

| 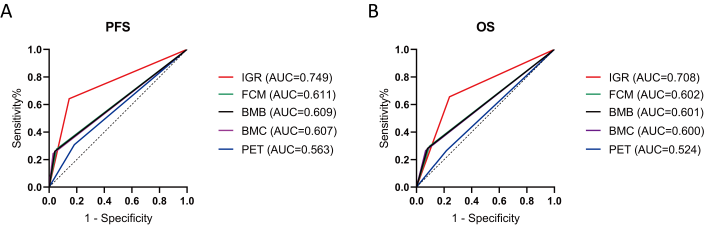 |
| --- |
| **Supplement Fig. 1.** Receiver operating characteristic (ROC) curves of all patients by different inspection methods according to the survival status assessed by (**A**) PFS; (**B**) OS. The Area under Curve (AUC) were calculated using the Mann-Whitney U test. |

| 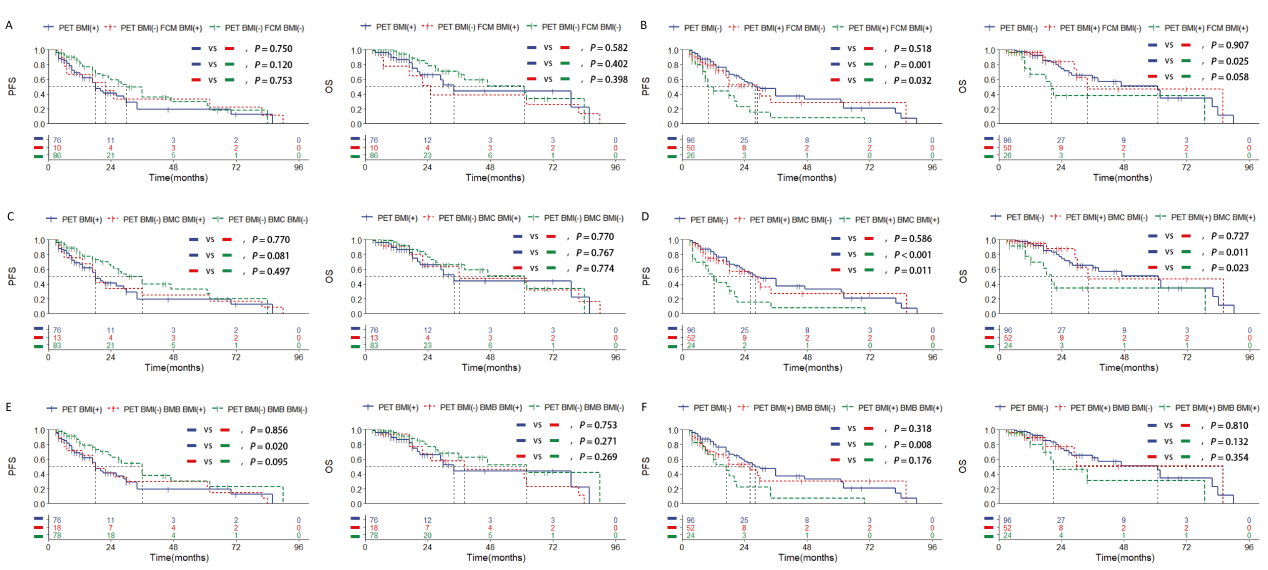 |
| --- |
| **Supplement Fig. 2.** Kaplan-Meier survival curves of patients of stage Ⅳ with DLBCL according to the BMI assessed by (**A, B**) combined assessment with PET/CT and flow cytometry; (**C, D**) combined assessment with PET/CT and bone marrow cytology; (**E, F**) combined assessment with PET/CT and bone marrow biopsy pathology. Survival panels present the PFS curves (left) and OS curves (right). Statistical differences were calculated using the log rank test. |

| 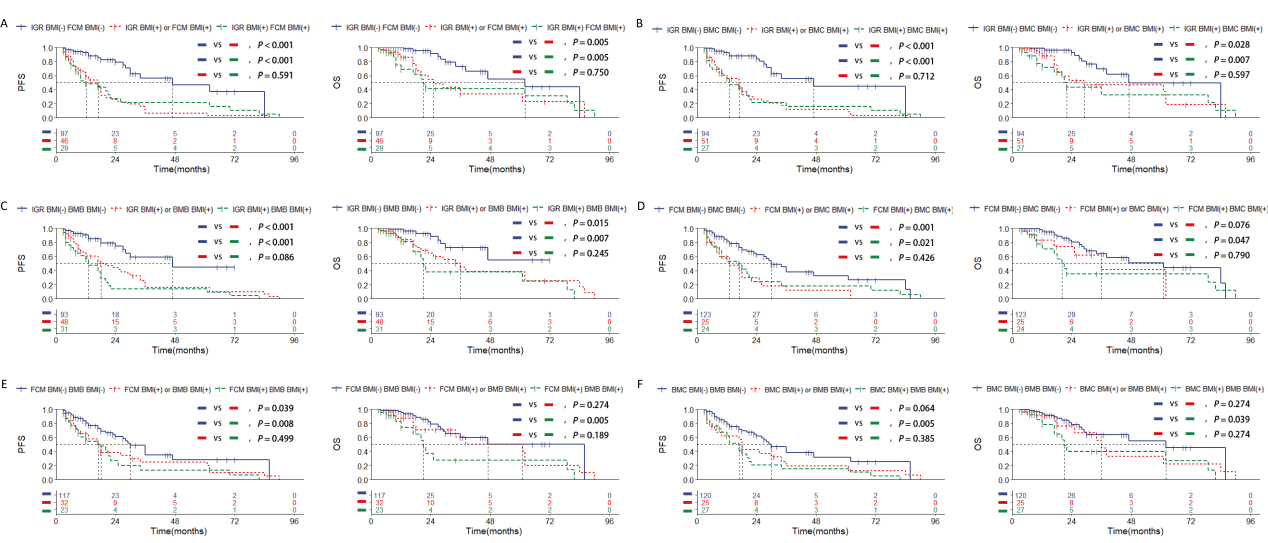 |
| --- |
| **Supplement Fig. 3.** Kaplan-Meier survival curves of patients of stage Ⅳ with DLBCL according to the BMI assessed by (**A**) combined assessment with immunoglobulin gene rearrangement and flow cytometry; (**B**) combined assessment with immunoglobulin gene rearrangement and bone marrow cytology; (**C**) combined assessment with immunoglobulin gene rearrangement and bone marrow biopsy pathology; (**D**) combined assessment with flow cytometry and bone marrow cytology; (**E**) combined assessment with flow cytometry and bone marrow biopsy pathology; (**F**) combined assessment with bone marrow cytology and bone marrow biopsy pathology. Survival panels present the PFS curves (left) and OS curves (right). Statistical differences were calculated using the log rank test. |

| 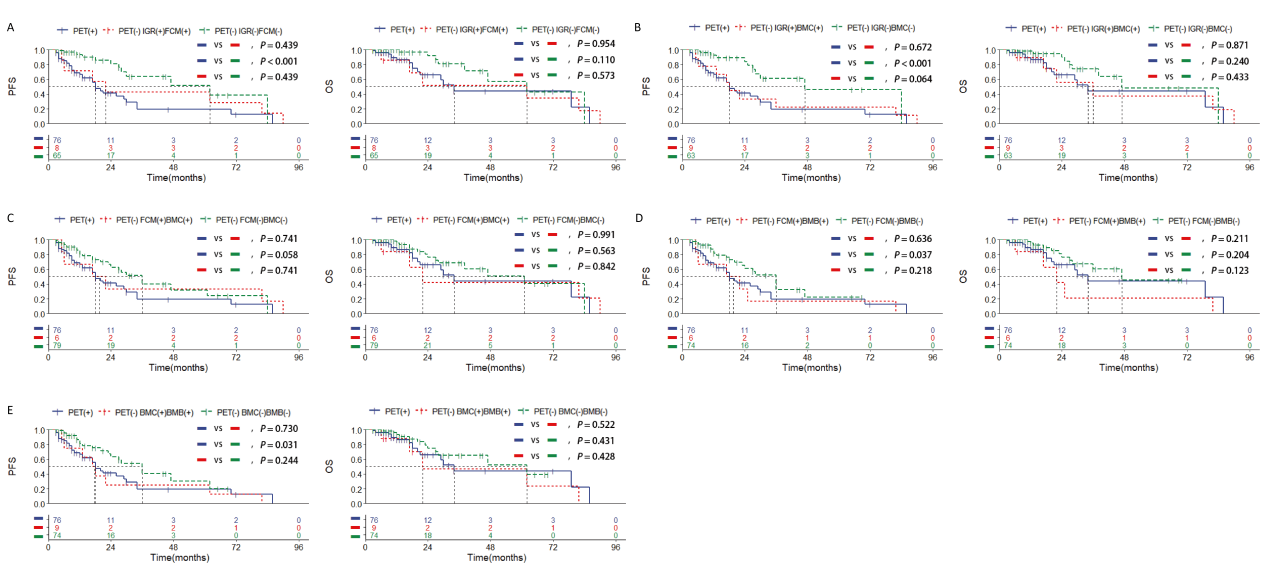 |
| --- |
| **Supplement Fig. 4.** Kaplan-Meier survival curves of patients of stage Ⅳ with DLBCL according to the BMI assessed by (**A**) combined assessment with PET/CT, immunoglobulin gene rearrangement and flow cytometry; (**B**) combined assessment with PET/CT, immunoglobulin gene rearrangement and bone marrow cytology; (**C**) combined assessment with PET/CT, flow cytometry and bone marrow cytology; (**D**) combined assessment with PET/CT, flow cytometry and bone marrow biopsy pathology; (**E**) combined assessment with PET/CT, bone marrow cytology and bone marrow biopsy pathology. Survival panels present the PFS curves (left) and OS curves (right). Statistical differences were calculated using the log rank test. |

| 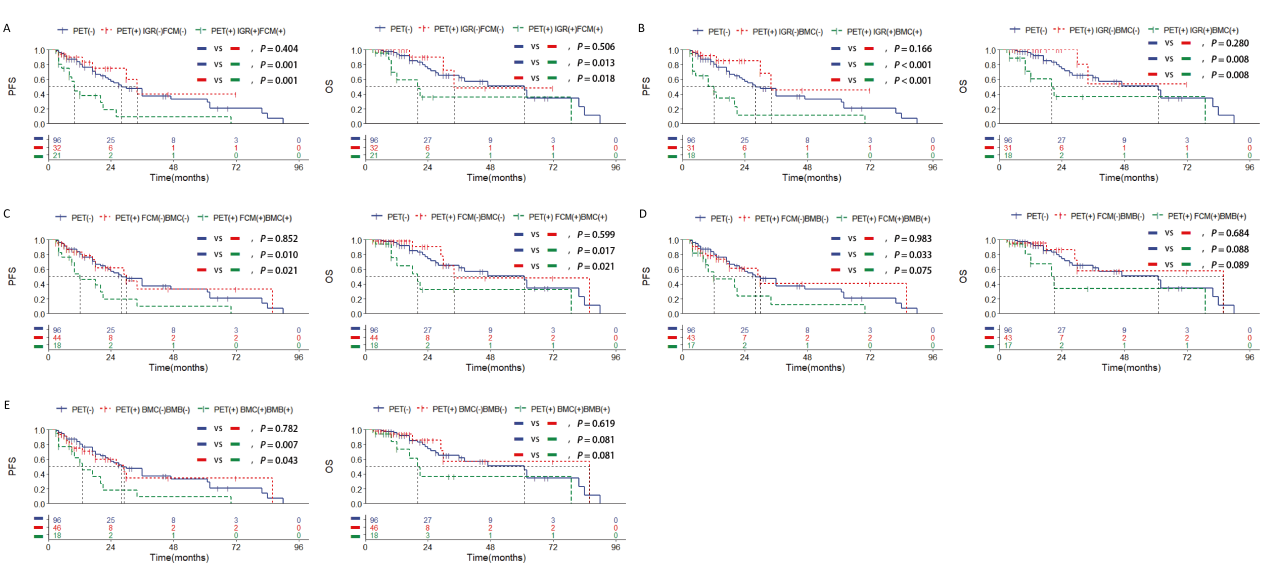 |
| --- |
| **Supplement Fig. 5.** Kaplan-Meier survival curves of patients of stage Ⅳ with DLBCL according to the BMI assessed by (**A**) combined assessment with PET/CT, immunoglobulin gene rearrangement and flow cytometry; (**B**) combined assessment with PET/CT, immunoglobulin gene rearrangement and bone marrow cytology; (**C**) combined assessment with PET/CT, flow cytometry and bone marrow cytology; (**D**) combined assessment with PET/CT, flow cytometry and bone marrow biopsy pathology; (**E**) combined assessment with PET/CT, bone marrow cytology and bone marrow biopsy pathology. Survival panels present the PFS curves (left) and OS curves (right). Statistical differences were calculated using the log rank test. |

| 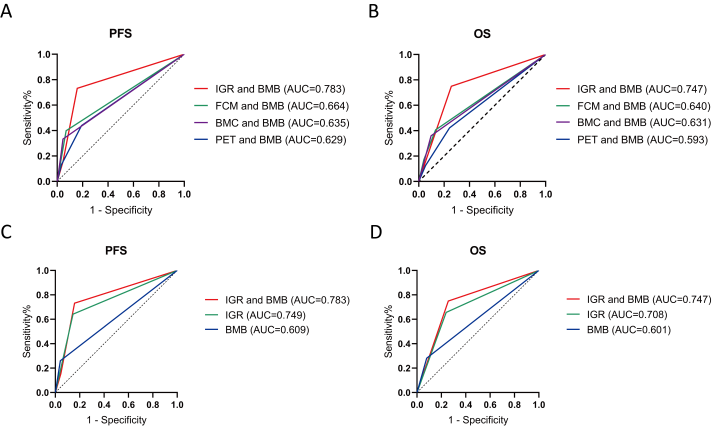 |
| --- |
| **Supplement Fig. 6.** Receiver operating characteristic (ROC) curves of all patients by different inspection methods according to the survival status assessed by (**A; C**) PFS; (**B; D**) OS. The Area under Curve (AUC) were calculated using the Mann-Whitney U test. |

| 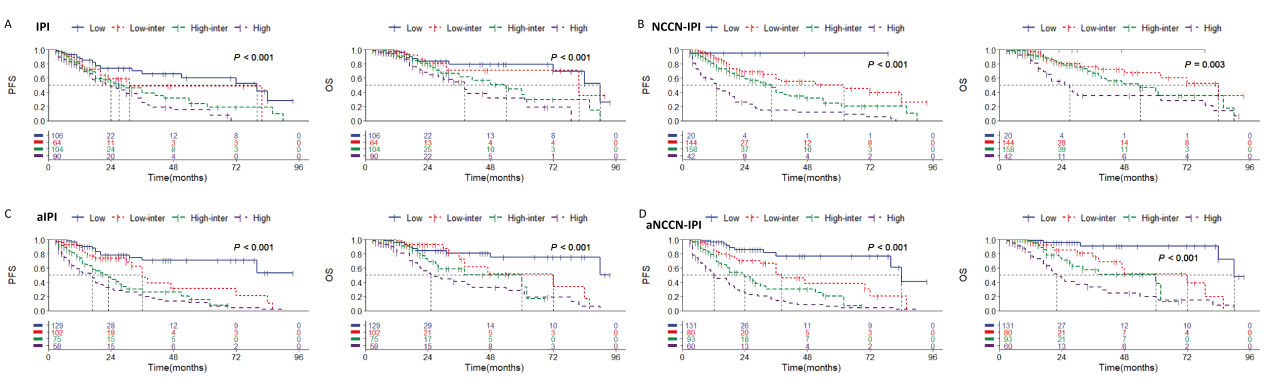 |
| --- |
| **Supplement Fig. 7.** Kaplan-Meier survival curves of patients of all patients with DLBCL according to (**A**) IPI; (**B**) NCCN-IPI; (**C**) Adjusted IPI; (**D**) Adjusted NCCN-IPI. Survival panels present the PFS curves (left) and OS curves (right). Statistical differences were calculated using the log rank test. |

| 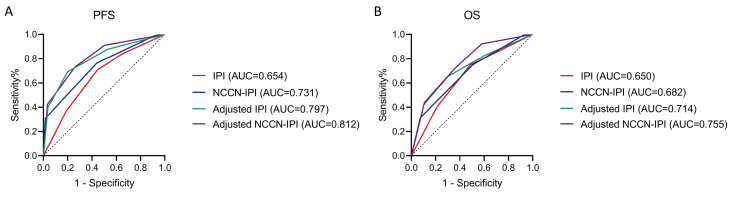 |
| --- |
| **Supplement** **Fig. 8.** Receiver operating characteristic (ROC) curves of all patients according to the survival status assessed by (**A**) PFS; (**B**) OS. The Area under Curve (AUC) were calculated using the Mann-Whitney U test. |
